# Phylogeography of the *Microcoleus vaginatus* (Cyanobacteria) from Three Continents – A Spatial and Temporal Characterization

**DOI:** 10.1371/journal.pone.0040153

**Published:** 2012-06-27

**Authors:** Petr Dvořák, Petr Hašler, Aloisie Poulíčková

**Affiliations:** Department of Botany, Palacký University Olomouc, Olomouc, Czech Republic; Barnard College, Columbia University, United States of America

## Abstract

It has long been assumed that cyanobacteria have, as with other free-living microorganisms, a ubiquitous occurrence. Neither the geographical dispersal barriers nor allopatric speciation has been taken into account. We endeavoured to examine the spatial and temporal patterns of global distribution within populations of the cyanobacterium *Microcoleus vaginatus*, originated from three continents, and to evaluate the role of dispersal barriers in the evolution of free-living cyanobacteria. Complex phylogeographical approach was applied to assess the dispersal and evolutionary patterns in the cyanobacterium *Microcoleus vaginatus* (Oscillatoriales). We compared the 16S rRNA and 16S-23S ITS sequences of strains which had originated from three continents (North America, Europe, and Asia). The spatial distribution was investigated using a phylogenetic tree, network, as well as principal coordinate analysis (PCoA). A temporal characterization was inferred using molecular clocks, calibrated from fossil DNA. Data analysis revealed broad genetic diversity within *M. vaginatus*. Based on the phylogenetic tree, network, and PCoA analysis, the strains isolated in Europe were spatially separated from those which originated from Asia and North America. A chronogram showed a temporal limitation of dispersal barriers on the continental scale. Dispersal barriers and allopatric speciation had an important role in the evolution of *M. vaginatus.* However, these dispersal barriers did not have a permanent character; therefore, the genetic flow among populations on a continental scale was only temporarily present. Furthermore, *M. vaginatus* is a recently evolved species, which has been going through substantial evolutionary changes.

## Introduction

Having been intensively studied over the past two decades, biogeography is one of the crucial factors necessary for an understanding of the ecological, evolutionary, and diversity patterns of prokaryotes [1], [2].

Generally, two different approaches toward the biogeography of free-living microorganisms have recently been discussed. (1) Historically, an older hypothesis claims that the occurrence of free-living organisms is driven by the environment, which selects the composition of a microbial community. The dispersal is then considered without any barriers (ubiquity); therefore, allopatry does not affect speciation [3], [4]. (2) To the contrary, some authors have recently advocated the existence of dispersal barriers and even endemic taxa within free-living microorganisms [2], [5]–[12]. The existence of some of the desmids' distributional areas resembling the phytogeographical patterns of vascular plant taxa has been noted by some authors [13], [14]. If the biogeography patterns of prokaryotes are closely related to those in eukaryotes [1], the existence of allopatric speciation can be expected [15].

The idea of cosmopolitanism is supported in some cyanobacteria by molecular markers, e.g. *Coleofasciculus* (*Microcoleus*) *chthonoplastes* [16], *Microcystis aeruginosa* [17]. However, van Gremberghe *et al.* [17] suggested the existence of a globally distributed population, which locally undergoes repeated events of bottleneck and selective sweeps [18], [19]. This drives speciation without any specific biogeographical pattern and allopatry. Arguments against ubiquity have recently been suggested in situations of geographical isolation on the continental level in thermophilic cyanobacteria such as *Synechococcus* spp. [20], *Mastigocladus laminosus* [21]. The inconsistency among the findings (mentioned above) implies a poor understanding of the overall mechanisms involved in cyanobacterial biogeography.

The cyanobacterium *Microcoleus vaginatus* (Vaucher) Gomont appears to be a suitable model organism for the evaluation of the biogeography and evolutionary patterns within free-living cyanobacteria, due to its world-wide distribution as well as its relatively easy identification, isolation, and culturing. *M. vaginatus* is an important primary producer within soil crusts and other subaerophytic environments all around the World.

[22]–[24]. However, *M. vaginatus* has also been isolated from freshwater epipelon [25], and from periodically dry puddles (this study); thus, indicating that it is not strictly aerophytic. Its taxonomy has been sufficiently studied [23], [26] and it has been genetically well characterized by the presence of an 11-bp insert in its 16S rRNA gene, which is a molecular autapomorphy for this species [22], [23]. However, practical identification of cultured strains is problematic because some important morphological features are missing in cultured materials, particularly the multiple filaments in a common sheath (e.g. [23]).

The 16S rRNA gene is a molecular marker, frequently used in the taxonomy and ecology of cyanobacteria, particularly on the genus level; additionally, there are a huge number of sequences available in GenBank (e.g. [27]). By contrast, 16S-23S ITS (internal transcribed spacer) is a variable region, which seems to be suitable for investigation on (and below) the species level, even for population genetics [28], [29].

Evolutionary relationships on different taxonomical levels are usually visualized graphically using phylogenetic trees. Nevertheless, when such mechanisms as recombination, horizontal gene transfer, or hybridization are taken into account, phylogenetic networks are more appropriate [30]. Accordingly, the network construction approach is also advantageous for the phylogeny of prokaryotic organisms (e.g. [31]).

The present study focuses on the evolutionary dispersal and distributional patterns of *M. vaginatus*, isolated from different continents, based on the 16S rRNA gene and 16S-23S ITS region, using phylogeographic methods combining both the tree and network, as well as PCoA analysis. Molecular clocks were applied in order to put the spatial distribution of *M. vaginatus* into a temporal framework.

## Materials and Methods

### Ethics statement

No specific permits were required for the described field studies. No specific permission was required for any locations and activity. The locations are not privately owned or protected in any way. No activity during field study involved any endangered species or protected species.

### Sample collection and cultivation

Altogether, 21 strains of *M. vaginatus* and 7 strains of *Phormidium* spp. (only used for the 16S rRNA analysis) were obtained either from natural samples or from the Culture Collection of Autotrophic Organisms (CCALA; http://www.butbn.cas.cz/ccala/index.php).

**Figure 1:**
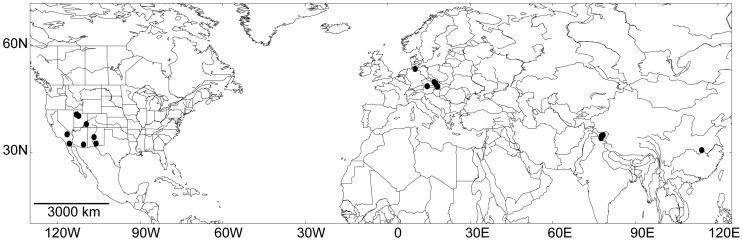
Location of *M vaginatus* sampling sites. The locations of the North American strains were adopted from Boyer *et al.* [23] and Siegesmund *et al.* [25].

The samples were collected from different habitats (e.g. puddles, moistened soil) and geographic sites (See Figure 1 and Table S1). Unialgal cultures were isolated following standard techniques [32]. The identification of all strains was based on their morphology using a light microscope, and following the system *sensu* Komárek & Anagnostidis [24]. The cultures were maintained in 100 mL Erlenmeyer flasks under the following conditions: temperature 22±1°C, illumination 20 µmol/m^2^/s, light regime: 12h light/12h dark, and liquid Zehnder medium [33].

### DNA extraction, PCR, and sequencing

Genomic DNA was extracted using an UltraClean Microbial DNA Isolation Kit (MOBIO, Carlsbad, CA, USA) from approximately 30 mg of fresh biomass, harvested during the log phase of the culture growth. 1.5% agarose gel, stained with ethidium bromide, was used to check DNA quality.

Partial 16S rRNA genes and the whole 16S-23S ITS region were PCR amplified using primers: forward P2 (5′-GGGGAATTTTCCGCAATGGG-3′), and reverse P1 (5′-CTCTGTGTGCCTAGGTATCC-3′). The combination of primers was previously described in Boyer *et al*. [23]. The PCR reaction, with a total volume of 20 µL, contained: 8.5 µL of sterile water, 0.5 µL of each primer (0.01 mM concentration), 10 µL FastStart PCR master (Roche Diagnostics GmbH, Mannheim, Germany), and 0.5 µL of template DNA (50 ng.µL^−1^). The PCR reaction was performed under the following conditions: initial denaturation for 4 min at 95°C, followed by 35 cycles of denaturation for 30 s at 95°C, annealing for 30 s at 57°C, extension for 1 min 50 s at 72°C, and lastly the reaction was completed with an extension for 7 min at 72°C. Quality PCR products (∼1600 bp) was examined on 1.5% agarose gels, stained with ethidium bromide. The PCR products, amplified from newly obtained strains, were cloned using a StrataClone PCR Cloning Kit (Agilent Technologies, Stratagene Product Division, La Jolla, CA, USA), following the manufacturer's instructions. After the white-blue selection on ampicillin 1.5% agarose plates with Luria Bertani medium, at least 4 positive colonies were transferred into fresh liquid Luria Bertani medium and cultured overnight at 37°C. The plasmid was isolated using a QIAGEN Plasmid Mini Kit (QIAGEN Inc., Valencia, CA, USA). The PCR product, amplified from culture collection strains, was purified using a GenElute^TM^ PCRClean-Up Kit (Sigma-Aldrich, Co., Saint Louis, MO, USA).

Both the plasmid (all positive clones) and purified PCR product were sent for commercial sequencing. The plasmids were sequenced using primers M13f and M13r, with the additional internal primers P5.

(5′-TGTACACACCGCCCGTC-3′), and P8 (5′-AAGGAGGTGATCCAGCCACA-3′), which have been previously described [23], [29]. The PCR products were sequenced using the same primers as used for amplification, with the additional internal primers P5 and P8 (see above). The sequences were assembled and proofread in a Sequencher 4.10 (Gene Codes Corporation, Ann Arbor, MI, USA); then they were deposited in GenBank (http://www.ncbi.nlm.nih.gov/). Accession numbers of the 16S rRNA sequences are JQ712618 to JQ712645, and 16S-23S ITS JQ712646 to JQ712666. All of the clones which were generated from each strain were aligned (ClustalX 2.0.11) [34]. All clones from all individual strains were found to be completely identical. Therefore, each strain is represented by one sequence.

### Phylogenetic and statistical analyses

The 16S rRNA Sequences were checked against chimeras and other anomalies within Mallard 1.02 software [35]. Multiple sequence alignment of both the 16S rRNA gene and 16S-23S ITS was performed by the ClustalW [34] algorithm, implemented in MEGA 5.05 [36], and corrected manually in a MEGA software alignment editor; following, were then exported in different formats for further analyses. The 16S-23S ITS sequences were used to construct the phylogenetic tree, as well as the network; further, to conduct the P-test and the PCoA analysis.

All available sequences, with their known geographical origin in GenBank (containing both genes tRNA^Ile^ and rRNA^Ala^ and with a known geographical origin) of *M. vaginatus* 16S-23S ITS, were added to the studied strains for analysis. Those sequences which had originated from desert soil crusts in the USA were well defined and had been previously published in Boyer *et al*. [23] and Siegesmund *et al*. [26]. Maximum likelihood and neighbour joining analyses were conducted in MEGA. Bayesian Information Criterion [37] was employed to achieve the most appropriate substitution model for maximum likelihood, and was determined as HKY+G (sample size: 647). The substitution model used in the neighbour joining analysis was the Kimura 2-parameter model [38]; with gaps treated as missing data. In both cases, bootstrap resampling was performed using 1000 replications.

A Neighbour-net phylogenetic network was constructed in SplitsTree4 4.11.3 [30], and all of the parameters were set at the defaults. The bootstrap test was performed using 1000 replications.

The Mantel test (9999 permutations) implemented in GenAlEx 6.4.1 [39] was performed in order to test the relationships between the geographic and genetic distances. The genetic distance matrix was inferred in MEGA, and the geographic distance matrix in GenAlEx 6.4.1.

A parsimony P-test [40] for strains which had originated from each continent, and the unweighted principal coordinate analysis (PCoA) were carried out in Fast UniFrac [41]. The best-scoring maximum likelihood tree, inferred in MEGA, was used for the input tree.

### Molecular clocks

The partial 16S rRNA gene was used to estimate the dates of divergence of *M. vaginatus*. Sufficiently long sequences (at least 1000 bp) with known geographical origins of *M. vaginatus* were selected from GenBank. Additional sequences from the entire spectrum of cyanobacteria (including partial 16S rRNA sequences of *Phormidium* spp. from the CCALA culture collection) were added to the analysis in order to achieve a broader taxonomic context, as well as more accurate results (total of 146 sequences). *Escherichia coli* was selected as the outgroup. To test the molecular clock hypothesis, a likelihood ratio test implemented in MEGA was used. The null hypothesis of equal substitution rates throughout the entire tree was rejected. Therefore, the relaxed uncorrelated clocks were selected for analysis [42]. The most suitable evolutionary model was presented using Bayesian Information Criterion [37] implemented in MEGA (sample size: 1010). The molecular clocks were calibrated based on the evolutionary distance between sequences of 16S rRNA obtained from fossil DNA samples and the closest recent descendant that could be identified using BLAST (http://blast.ncbi.nlm.nih.gov/Blast.cgi).

All clones (16S rRNA fragments isolated from a 5.8–5.9 Ma late Miocene gypsum crystals) except the two presented in Panieri *et al*. [43] were used. One of the excluded clones was not determined in the study, as there is no sequence deposited in GenBank (see [43]). The second (FJ809895) had the most related recent descendant among eukaryotic chloroplasts. A pairwise distance (in substitutions per site) between each ancestor/descendant sequences was calculated using p-distance model in MEGA. Subsequently, the final substitution rate per site per million years was determined as the mean of all individual pairwise distances per million years. The standard deviation and 95% confidence interval (CI) were calculated. Specific values are shown in the Table S2. The mean substitution rate per million years (0.001861) and 95% CI (0.000643–0.003079) with uniform distribution was set for further analysis, carried out in BEAST 1.6.1 [44]. The analysis was set with the following parameters: GTR+G+I substitution model, MCMC chain length of 6.0004×10^7^ generations, sampled each 1.4×10^4^ generation, and relaxed uncorrelated lognormal clock [42]. The BEAST.xml file was created in BEAUTi [44]. Due to the temporal demands of the computation, the analysis was carried out on the web portal CIPRES Science Gateway (specialized in phylogeny), where BEAST is implemented [45]. The effective sample size (ESS) was evaluated using TRACER 1.5 [46]. The final maximum credibility tree was annotated using TreeAnnotator 1.6.1 [44], with the first 100 trees burned-in.

## Results

### Species identification

All of the strains that were under investigation showed the characteristic features according to Komárek & Anagnostidis [24]. *M. vaginatus* strains CCALA 757, 143, and 152 had originally been incorrectly identified and assigned as different species of the genus *Phormidium* within the culture collection. Our re-identification to *M. vaginatus* is based on light microscopy morphology as well as the presence of 11-bp insert within the 16S rRNA. All of the strains were coherent in their important morphological characteristics (cell dimension, shape, cell division, and the presence of calyptra).

### 16S-23S ITS phylogeographical analysis

Altogether, 32 sequences obtained from strains having originated from three continents (Europe, Asia, and North America) were analysed using two phylogenetic approaches (tree and network), and PCoA analysis. All 16S-23S ITS sequences contained both genes for tRNA^Ile^ and rRNA^Ala^; therefore, the dataset did not exhibit large gaps which possibly could negatively influence the results. The Mantel test showed a very significant correlation between the geographic and genetic distances (R = 0.184, P = 0.0001).

**Figure 2:**
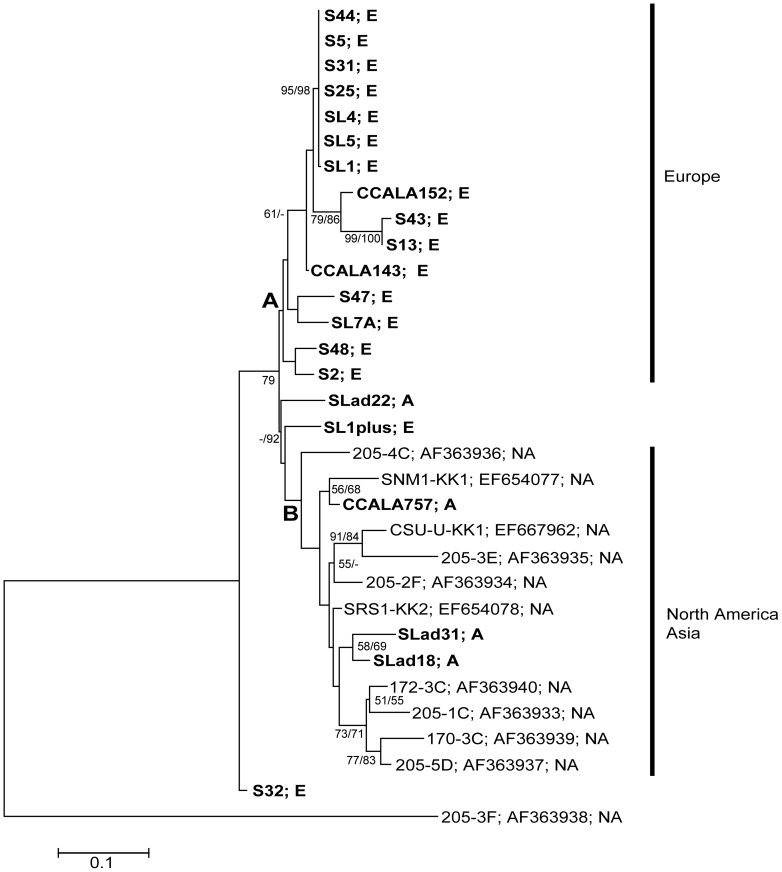
Maximum likelihood inferred phylogenetic tree based on the 16S-23S ITS of *M. vaginatus*. Maximum likelihood/neighbour joining bootstrap supports greater than 50% are shown at the nodes. The studied strains are in bold. The geographical origin of each strain is indicated as E – Europe, A – Asia, and NA – North America.

The maximum likelihood tree (MEGA) revealed two clades: (A) European *M. vaginatus*, and (B) North American and Asian strains (Figure 2). Therefore, the European strains were distinguishable from the North American and Asian, with the exception of two strains with a transitional position between both clades (strains SLad22 and SL1plus, Figure 2). However, the North American and Asian strains clustered together within clade B, without any particular biogeographical pattern. Both clades (A and B) included a couple of subclades (diversified genotypes), without any respect to the autecology of the strains. Strains S32 and 205-3F had an uncertain position within the tree, without any significant bootstrap support. Internal nodes within both clades A and B (Figure 2) had good bootstrap support; however, the clades themselves were very poorly supported. Thus, a phylogenetic network and the PCoA analysis approach were employed in order to achieve more accurate results.

**Figure 3:**
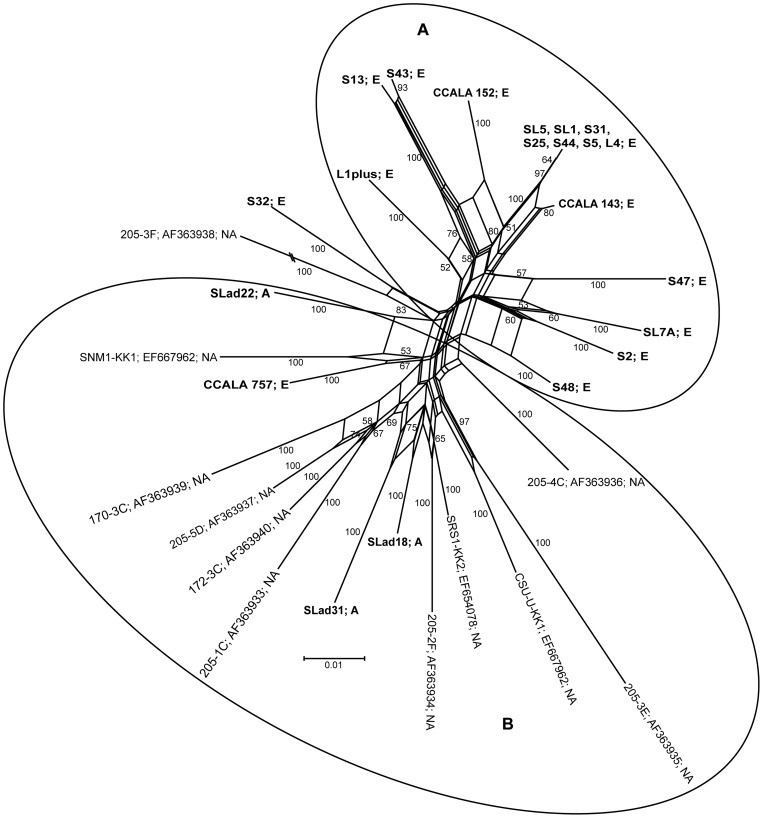
Neighbour-net phylogenetic network based on the 16S-23S ITS of *M. vaginatus.* Bootstrap supports greater than 50% are indicated. The studied strains are in bold. The geographical origin of each strain is indicated as E – Europe, A – Asia, and NA – North America.

The almost identical topology showed a neighbour-net network constructed using SplitsTree (Figure 3). The network exhibited groups A (European) and B (North America and Asian, Figure 3), containing almost the same taxa as did the phylogenetic tree. The problematic strains SLad22 and SL1plus (see above) belonged to groups of their biogeographical origin, with high bootstrap support. The position of strains S32 and 205-3F was better resolved. However, strain 205-3F also exhibited a very long branch, suggesting its enormous distance from the other strains.

**Figure 4:**
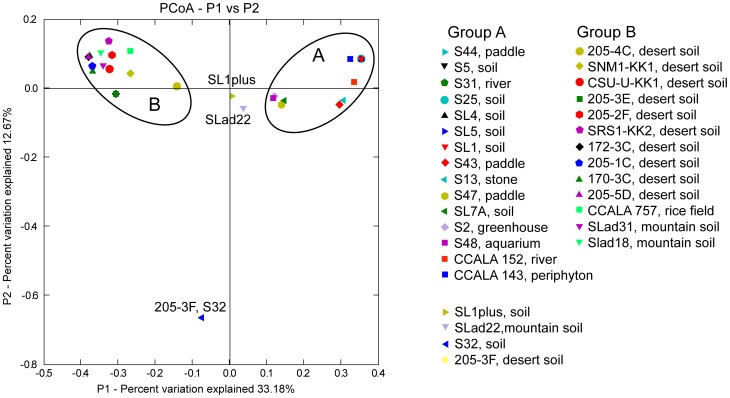
Principal coordinate analysis performed in Fast UniFrac based on the 16S-23S ITS of *M. vaginatus.* Principal coordinate 1 (P1) versus Principal coordinate 2 (P2) is shown. Group A consists of European strains, and group B of North American and Asian.

A similar grouping pattern revealed the PCoA analysis carried out in Fast UniFrac (Figure 4) where the habitat type was taken into account. European strains (group A) formed a separate group from those strains which had originated from North America and Asia (Group B), without any respect to habitat type. Strains SLad22, SL1plus, S32, and 205-3F showed uncertain positions similar to the phylogenetic tree and network.

The clustering of strains in the phylogeny (tree, network) and PCoA analysis were also confirmed by corrected P-values (Fast UniFrac) for those strains isolated from individual continents. European strains were significantly different from the North American and Asian (P≤0.002), and the difference between the North American and Asian were only marginally significant (P≤0.096).

All analyses suggest that strains of *M. vaginatus* which originated from the Europe are genetically different from those isolated from North America and Asia. Therefore, a dispersal barrier between Europe and Asia might well exist; with the speciation of these cyanobacteria also being driven by their geographical isolation. On the other hand, very close relationships, accompanied by an uncertain dispersal pattern between the North American and Asian strains, suggests frequent genetic exchanges between *M. vaginatus* populations on these two continents.

### Divergence dating estimation

The dating analysis of the 16S rRNA gene in BEAST was calibrated at an evolutionary rate of 0.001861 substitutions per site per million years (95% CI = 0.000643–0.003079), which has only recently been determined for cyanobacterial 16S rRNA by the comparison of fossil and recent 16S rRNA sequences (see Materials and Methods). This approach gives a coherent image of the divergence times among recent living cyanobacteria; this is because there is a lack of convincing calibrating points and dissimilar substitution rates among the different groups of bacteria [47].

**Figure 5:**
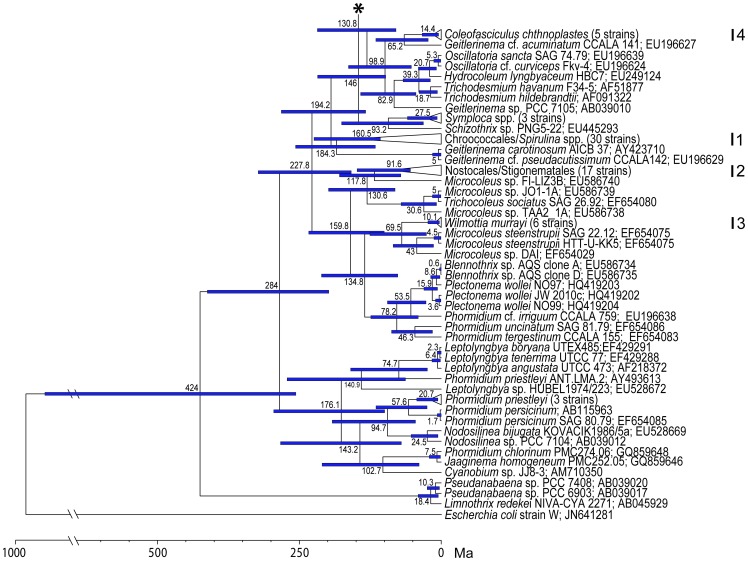
The dating of the divergence times among cyanobacteria. Maximum credibility chronogram based on 16S rRNA of cyanobacteria, with *Escherichia coli* as an outgroup. The mean ages and confidence intervals (95% HPD) are indicated at the nodes. An asterisk represents a node where Figure 5 and 6 were originally connected.

**Figure 6:**
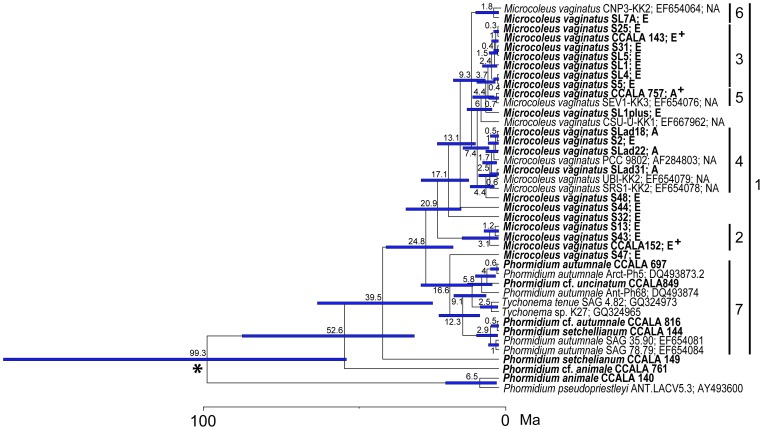
The dating of the divergence times among cyanobacteria. Maximum credibility chronogram based on 16S rRNA of cyanobacteria, with an emphasis on *Microcoleus vaginatus*. It is a continuation of Figure 5. The mean ages and confidence intervals (95% HPD) are indicated at the nodes. The studied strains are in bold. The geographic origin of each strain is indicated as E – Europe, A – Asia, and NA – North America. A plus mark indicates the strains which have been identified anew because of previous incorrect determinations in the culture collection. An asterisk represents a node where Figure 5 and 6 were originally connected.

The chronogram (Figure 5, 6), based on 16S rRNA, shows divergence times within all groups of cyanobacteria (Chroococcales, Oscillatoriales, Nostocales, and Stigonematales *sensu* Komárek & Anagnostidis [24]); however, focused on *M. vaginatus* (Figure 6). Recent unicellular cyanobacteria (order Chroococcales) diverged from Oscillatoriales before 184.3 Ma, 95% HPD (highest posterior density interval) 116.1–256.6 (clade 1, Figure 5), and formed a monophyletic group with the exception of two filamentous cyanobacteria *Spirulina* spp. Some of recent heterocystous cyanobacteria (Nostocales and Stigonematales; clade 2, Figure 5) diversified one time before 117.8 Ma (95% HPD 71.1–179.3) from filamentous cyanobacteria and formed a monophyletic group (clade 2, Figure 5).

*M. vaginatus* separated from the other filamentous cyanobacteria (order Oscillatoriales) before 39.5 Ma (95% HPD 22.5–61.8; clade 1, Figure 6) and formed a monophyletic clade with *Phormidium autumunale*/*Tychonema* spp., which is its sister clade 7 (Figure 6). The European strains were concentrated in clades 2 and 3; moreover, they formed other individual lineages of strains SL1plus, S48, S44, S32, and S47. Thus, the European strains have been derived at least twice: clade 2 (20.9 Ma; 95% HPD 12.8–31.6), and clade 3 (3.7 Ma; 95% HPD 1.2–7.5). On the other hand, clade 4 was composed of strains having originated from Asia, North America, and the S2 European strain, and diverged sometime before 4.4 Ma (95% HPD 1.5–9.8). Clade 5 was composed of two strains which originated from Asia and North America, having originated before 3.7 Ma (95% HPD 1.2–7.5). Similarly, Clade 6 included one North American and one European strain which originated before 9.3 Ma (95% HPD 4.6–15.5). Clade 7 (Figure 6) *Phormidium autumnale/Tychonema* spp. diverged before 16.6 Ma (95% HPD 8.8–26.5).

*M. vaginatus* appears to have diversified later than the other Oscillatoriales species. For instance, the newly described *Wilmottia murrayi* [48] (clade 3, Figure 5) diverged before 69.5 Ma (95% HPD 26–125.5) and *Coleofasciculus chthnoplastes* [26] (clade 4, Figure 5) before 65.2 Ma (95% HPD 22.9–115.2).

The 16S rRNA based tree exhibits a similar branching pattern for *M. vaginatus* as does the 16S-23S ITS based tree, network and PCoA analysis. European strains retained a geographical separation from North American and Asian strains. However, some minor discrepancies appeared. The European strains formed two separate clades with North American and Asian strains in between them (see details above). The North American and Asian strains showed a close phylogenetic relationship in all performed analyses. Some strains formed individual lineages, e.g. S48, S44 and S47. The position of these strains was better resolved in the 16S-23S ITS phylogeny, where they clustered together with the other European strains (Figure 2, 3, 4, group A). The European strains S2 and SL7A nested among the North American and Asian (clades 4–6, Figure 6) in comparison with the 16S-23S ITS phylogeny, where they retained the cluster of their geographical origin (Figure 2, 3, 4, group A). This suggests that in combination with the relatively long temporal distances among clade divergences the isolation of populations on a continental scale may have a temporary character.

## Discussion

Although *M. vaginatus* is a common cyanobacterium that is distributed worldwide, its biogeography and possible dispersal patterns have yet to be sufficiently studied on a large scale. The species seems to be “cosmopolitan” and “ecologically euryvalent”, inhabiting aerophytic and freshwater habitats (Table S1). A similar situation has been found with many other microalgae, which were also considered to be cosmopolitan before their cryptic diversity had been described [49]–[53]. Because microalgal speciation is not always accompanied by morphological change, the true number of species is likely to be greater than the current tally of nominal species, most of which are delineated on purely morphological grounds [54]. Previously, *M. vaginatus* had also been suggested as a complex, composed of several cryptic species [23], [26]. However, *M. vaginatus* has diverged rather recently in comparison with the other species of filamentous cyanobacteria (e.g. *Wilmottia murrayi, Colofasciculus chthnoplastes*, Figure 5, clade 3, 4). It has been undergoing significant evolutionary differentiations, both spatial and temporal.

Recently, several species concepts have been proposed, applicable to the cyanobacteria. All of them treat the question of cosmopolitanism and endemism differently. The Evolutionary Species Concept describes a species as an entity, composed of organisms, which has its own historical and future evolutionary tendencies [55]. *M. vaginatus* would then possess several separate evolutionary lineages (Figure 2), each being characterized by geographic origin, as well. Thus *M. vaginatus* would not be considered as cosmopolitan. The Ecotypic Species Concept *sensu* Cohan [18] defines species (ecotype) based upon its ecological niche. Phylogenetic analysis (Figure 2) revealed various compositions of ecological features within a majority of the clades. Therefore, a true ecotype cannot be well defined. Johansen & Casamatta [56] proposed a modified Monophyletic Species Concept: species is a monophyletic clade, characterized by a unique apomorphy. There was no significance identified from either the morphological or molecular apomorphy for any particular clade. All of the studied strains only possessed their common synapomorphy (11-bp insert in 16S rRNA) [22], [23]. Although there is a considerable genetic variability among different populations, we assume that *M. vaginatus* is an immature species, in the early stages of evolution, and that the existence of cryptic species is still unclear.

The relationships for some microalgae to their ecological preferences [14] have not been confirmed in this study for *M. vaginatus*; however, this does not mean that ecology does not have any influence. Unfortunately, specific ecological data are only available for our isolates, not for most of the sequences obtained from GeneBank. Thus, the only “ecological parameter” used in this study is the biotope/habitat type. Both European and Asian strains originated from different biotopes (soil, puddles, and river; see Table S1 for details), The American strains have only been isolated from desert crusts [23]. Although strains from both clusters differ ecologically, they did not exhibit any particular clustering patterns, dependent on habitats (Figure 2, 3, 4). For example, strain CCALA 757 (isolated from a rice field) was very close to strain SNM1-KK1 (isolated from desert crust). Indian strains (SLad 18, 22, and 31) were isolated from soil crusts in the Ladakh (Himalaya), where the average annual temperature is around −8.2°C [57]. Therefore, we assume that the clustering pattern within the tree, network, P-test, and PCoA analysis (Figure 2, 3, 4) is more likely the result of geographic differentiation, and not from the strains' autecology. This is also confirmed by the very significant correlation between the geographic and genetic distances in the Mantel test (R = 0.184, P = 0.0001), which should be a relevant support for the existence of phylogeography among the strains of *M. vaginatus*.

The aerophytic and subaerophytic habitats are optimal biotopes for studying the biogeographical and dispersal patterns of free-living microorganisms on the continental scale, since there are large potential barriers, which may prevent dispersal. Taton *et al*. [6] proposed the endemism of some Antarctic cyanobacteria investigated, combining morphology and analysis of the 16S rRNA. Similarly, Miller *et al*. [21] and Papke *et al*. [20] found dispersal barriers among extremophilic cyanobacteria. Later, Jungblut *et al*. [58] argued for the cosmopolitanism of cyanobacteria within the Polar Regions, having investigated large numbers of 16S rRNA sequences, and having found up to a 99.9% similarity among some individual Arctic and Antarctic isolates. Analysis of the polar *Phormidium autumnale* revealed an identical image [59]. Gracia-Pichel *et al*. [22] suggested a cosmopolitan occurrence of *M. vaginatus*, without any dispersal barriers. However, this statement was based on six 16S rRNA sequences as well as DGGE analysis. Our data showed geographical differentiation among *M. vaginatus*, which originated from different continents. The European strains differed from those which originated from North America and Asia. Surprisingly, the North American and Asian strains showed a very high similarity among themselves (Figure 2, 3, 4). This suggests that there were a greater genetic flow between the American and Asian populations. A possible explanation for this phenomenon is indicated by the global system of dust transport, where large regular dust flows are directed from Asian to the American deserts [60]. However, the European strains do not seem to be fully isolated. There appear some transitions such as strain S32, which may indicate that this particular strain is the result of a newly evolved genotype.

The mechanisms of speciation in prokaryotes differ from those in eukaryotes. Prokaryotic organisms do not exhibit sexual reproduction; they have extremely large populations and high dispersal abilities, small sizes of the individual, and the ability to produce resting stages. Therefore, the most important speciation mechanisms are considered horizontal gene transfer, homologous recombination, and periodic selection. Allopatry (geographical isolation) is not predominantly regarded as a crucial factor [17], [61]–[63]. Our results revealed that *M. vaginatus* has certain dispersal barriers on the continental level. Thus, we suggest that allopatry is also an important speciation factor in *M. vaginatus*, although geographical isolation may only have a temporary character. This will be discussed further.

Divergence dating analysis (Figure 5, 6) uncovered unique evidence of temporal characterizations of *M. vaginatus*'s evolutionary and dispersal patterns. The chronogram revealed that recent European strains have diverged more than once, and that there were significantly long periods of time between events. Because of these long periods of time, we assume that while dispersal barriers existed, the gene flow among populations from Europe to other continents was not continuous. North American and Asian populations appear to have diverged almost simultaneously; additionally, there were no particular dispersal patterns found (Figure 5, 6). Furthermore, dispersal of *M. vaginatus* does not seem to be dependent on continental drift, because the differentiation of the genotypes took place after the division of Euroasia and America, which occurred during the Cretaceous [64].

The molecular clocks for prokaryotes may be inferred based upon the fossil records, host fossil records, associations with ecological events, or molecular clocks derived from eukaryotes [65]. Because there is lack of convincing calibrating points, as well as significant differences among substitution rates within prokaryotes [47], we inferred a novel substitution rate for the cyanobacterial 16S rRNA gene from fossil DNA. Ochman & Wilson [66] proposed the universal 16S rRNA evolutionary rate of 1% change per 50 million years for bacteria. Moran *et al*. [67] suggested rates of 1–2% per 50 million years, from the relationships of aphids and its endosymbiont. Both of these universal calibrations ticked significantly slower than the rate determined in this study. One probable explanation is that these aforementioned rates were calculated for groups of bacteria other than cyanobacteria, which have unique physiological and ecological features among the other prokaryotes [68].

Our results show that dispersal barriers have played an important role in the evolution and ecology of *M.* vaginatus on the global scale; therefore, the speciation of *M. vaginatus* is also affected by allopatry. However, these dispersal barriers do not have a permanent character.

## Supporting Information

Table S1 List of investigated strains.
(DOC)

Table S2 Identified evolutionary rates.
(DOC)
