# Supplementary material for: Phylogeography of the Microcoleus vaginatus (Cyanobacteria) from Three Continents – A Spatial and Temporal Characterization
Source: PLoS One. 2012 Jun 27;7(6):e40153. doi: 10.1371/journal.pone.0040153 (PMC3384635; doi:10.1371/journal.pone.0040153)
Supplement: Table S1 — List of investigated strains. (DOC) [file pone.0040153.s001.doc]

**Table S1** List of investigated strains, their location, and habitat of origin. Strains marked with an asterisk were previously assigned to another species in the CCALA Culture Collection (http://www.butbn.cas.cz/ccala/index.php); see the CCALA website for details. Some specifications in the culture collection which were not available the CCALA are marked with NA.

| Strain | GPS coordinates | Locality | Habitat |
| --- | --- | --- | --- |
| *M. vaginatus* SLad22 | 34°39'02.9''N, 077°41'17.0''E | Gompa valley, Ladahk, India | soil crust |
| *M. vaginatus* Slad31 | 34°02'32.5''N, 077°31'37.4''E | Stok valley, Ladahk, India | soil crust |
| *M. vaginatus* Slad18 | 32°55'25.1''N, 078°14'30.0''E | Mentog, Ladahk, India | soil crust |
| *M. vaginatus* S32 | 48°39'17.733"N, 14°21'55.339"E | Rožmberk nad Vltavou, Czech Republic | soil |
| *M. vaginatus* SL1plus | 49°50'25.977"N, 16°54'4.152"E | Zvole, Czech Republic | soil |
| *M. vaginatus* SL7A | 49°50'25.977"N, 16°54'4.152"E | Zvole, Czech Republic | soil |
| *M. vaginatus* S2 | 49°34'31.462"N, 17°16'45.779"E | Olomouc, Czech Republic | greenhouse |
| *M. vaginatus* SL5 | 49°50'25.977"N, 16°54'4.152"E | Zvole, Czech Republic | soil |
| *M. vaginatus* SL1 | 49°50'25.977"N, 16°54'4.152"E | Zvole, Czech Republic | soil |
| *M. vaginatus* S31 | 48°39'17.733"N, 14°21'55.339"E | Rožmberk nad Vltavou, Czech Republic | river bottom sediment |
| *M. vaginatus* S44 | 49°35'46.874"N, 17°15'38.746"E | Olomouc, Czech Republic | puddle |
| *M. vaginatus* S5 | 49°38'44.095"N, 17°12'3.125"E | Horka nad Moravou,Czech Republic | soil |
| *M. vaginatus* SL4 | 49°50'25.977"N, 16°54'4.152"E | Zvole, Czech Republic | soil |
| *M. vaginatus* CCALA 143* | NA | Topolčany, Slovakia | periphyton |
| *M. vaginatus* CCALA 757* | NA | Wuhan city, China | rice field |
| *M. vaginatus* CCALA 152* | NA | Hamburg, Germany | river |
| *M. vaginatus* S43 | 49°34'40.627''N, 17°16'44.115"E | Olomouc, Czech Republic | puddle |
| *M. vaginatus* S47 | 49°44'34.584"N, 17°26'55.305"E | Domašov nad Bystřicí, Czech Republic | puddle |
| *M. vaginatus* S25 | 49°23'13.613"N, 17°42'48.681"E | Chvalčov, Czech Republic | forest soil |
| *M. vaginatus* S48 | 49°14'28.396"N, 17°28'12.207"E | Kvasice, Czech Republic | aquarium |
| *M. vaginatus* S13 | 49°16'21.255"N, 17°31'11.082"E | Stone-pit Kurovice | stone |
| *Phormidium autumnale* CCALA 697 | NA | Ellsemere island, Canada | NA |
| *P.* cf. u*ncinatum* CCALA 849 | 69°21´N, 18°49´E | Abisko, Sweden | wettened rock |
| *P. setchellianum* CCALA 144 | NA | Tuffewies, Switzerland | experimental trough |
| *P.* cf. a*utumnale* CCALA 816 | 69°21'N, 18°49'E | Abisko, Sweden | NA |
| *P.* cf. *setchelianum* CCALA 149 | NA | Hamburg, Germany | periphyton |
| *P.* cf. *animale* CCALA 761 | NA | Třeboň, Czech Republic | periphyton |
| *P. animale* CCALA 140 | NA | Vulcano Vesuv, Italy | soil |
