# Supplementary material for: Phylogeography of the Microcoleus vaginatus (Cyanobacteria) from Three Continents – A Spatial and Temporal Characterization
Source: PLoS One. 2012 Jun 27;7(6):e40153. doi: 10.1371/journal.pone.0040153 (PMC3384635; doi:10.1371/journal.pone.0040153)
Supplement: Table S2 — Identified evolutionary rates. (DOC) [file pone.0040153.s002.doc]

**Table S2** Substitution per site per million years identified for all investigated clones from Panieri et al. [43] used for subsequent calibration of the chronogram.

| Accession numbers from Panieri et al. [43] | Recent descendants’ accession numbers | Substitution per site per million years |
| --- | --- | --- |
| FJ809900 | FJ410907 | 0.00154 |
| FJ809896 | JQ580111 | 0.00051 |
| FJ809897 | JN216801 | 0.00068 |
| FJ809901 | JN813692 | 0.00017 |
| FJ809902 | EF522222 | 0.00154 |
| FJ809903 | AY712240 | 0.00154 |
| FJ809904 | DQ914863 | 0.00085 |
| FJ809905 | AY712240 | 0.00154 |
| FJ809898 | HQ197684 | 0.00478 |
| FJ809899 | AY274618 | 0.00546 |
| Mean |  | 0.001861 |
| Standard deviation |  | 0.001703 |
| 95% confidence interval |  | 0.000643–0.003079 |
